# Kick Cat Effect: Social Context Shapes the Form and Extent of Emotional Contagion

**DOI:** 10.3390/bs13070531

**Published:** 2023-06-26

**Authors:** Ling Zhang, Ying Chen, Yanqiu Wei, Jie Leng, Chao Kong, Ping Hu

**Affiliations:** Department of Psychology, Renmin University of China, No. 59 of Zhongguancun Street, Haidian District, Beijing 100872, China; ymtxlc@ruc.edu.cn (L.Z.); 2019000534@ruc.edu.cn (Y.C.); weiyanqiu@ruc.edu.cn (Y.W.); lengjie2021@ruc.edu.cn (J.L.); kongchao97@ruc.edu.cn (C.K.)

**Keywords:** emotional aggregation, emotional transmission, social appraisal, social context, facial expression

## Abstract

Emotional contagion refers to the transmission and interaction of emotions among people. Researchers have mainly focused on its process and mechanism, often simplifying its social background due to its complexity. Therefore, in this study, we attempt to explore whether the presence and clarity of social context affect emotional contagion and the related neural mechanisms. In Experiment 1, participants were asked to report their subjective experiences after being exposed to the facial expressions of emotional expressers, with or without the corresponding social context being presented. The results revealed that positive or negative expressions from the expressers elicited corresponding emotional experiences in the receivers, regardless of the presence of social context. However, when the social context was absent, the degree of emotional contagion was greater. In Experiment 2, we further investigated the effect of the clarity of social contexts on emotional contagion and its neural mechanisms. The results showed an effect consistent with those in Experiment 1 and highlighted the special role of N1, N2, P3, and LPP components in this process. According to the emotions as social information theory, individuals may rely more on social appraisal when they lack sufficient contextual information. By referencing the expressions of others and maintaining emotional convergence with them, individuals can adapt more appropriately to their current environment.

## 1. Introduction

Emotional contagion is a normal occurrence in everyday life, and it can spread among people like a yawn. Research has shown that emotional contagion is a process through which individuals capture the emotional state of others [1] and transmit emotions from one individual to another [2]. The process is always inspired by the expresser and eventually aligns the recipient’s emotions with the emotional experience of the expressers [3]. Generally, positive emotional contagion or moderated negative emotional contagion can promote relationships and cooperation.

During the process of emotional contagion, for the emotional recipient, although the expression of the expresser is the main emotional source leading to the occurrence of emotional contagion [4], the social context in which it occurs may also affect the incidence and degree of emotional contagion [5,6], particularly when facial expressions are ambiguous or the depicted emotional intensity is low [4]. Researchers define context as “information surrounding events” [7] and consider it to encompass the social and physical environment in which interpersonal communication takes place [4]. Moreover, context information plays a critical role in emotional perception [8,9] and the construction of emotional meaning [10,11] in social communication. Since the meaning of facial expression is inherently vague, it can only acquire a concrete meaning through interpretation, and this process largely relies on the context in which it occurs [12,13]. For instance, in the absence of a social context, a smile is merely an expression of affinity [14]. However, it may also serve as a cue for contempt or threat in a hostile context.

However, previous studies on emotional contagion do not focus on the importance of social context. Instead, they place greater emphasis on the characteristics of the expresser or receiver, such as eye orientation [15], face attractiveness [16], and so on, or on the relationship between the expresser and the receiver, such as intergroup relations [17,18]. As mentioned by Hess, social context is often excluded as an experimental distractor in these studies [9]. For instance, subjects are presented with carefully cropped images in advance, showing only facial parts [15,17], or even elliptical faces without hair [15,19,20], in order to minimize contextual information. However, we believe it is necessary to explore the influence of social context on emotional contagion. This is because the occurrence of emotional contagion is inherently tied to social context, and removing the social context would result in the absence of the complete background of emotional contagion, making it difficult to generalize the research findings to a broader scope. Moreover, social context can provide essential information for the occurrence of emotional contagion, such as whether the environment is safe and whether the emotional response is appropriate. Consistent with our viewpoint, Fischer et al. (2003) also mentioned in their relationship model between emotion and social context that the events triggering emotions and the social context in which emotional events occur are equally important. Social contexts and emotional events are often appraised together by individuals, thus becoming part of individual emotional responses [21].

The importance of social context in emotional contagion has also been observed in some previous studies. For instance, research on primitive emotional contagion has shown that emotional receivers automatically imitate the expressions of the expresser in a cooperative context, whereas they inhibit the tendency for emotional imitation in a competitive context [22,23,24]. Studies have further demonstrated that context can reshape emotional imitation [25]. For example, if someone smiles at you with hostile intentions, it may induce an anti-emotional contagion. It is the context that imbues emotional expressions with genuine intention [26], ultimately determining whether emotional contagion occurs.

In particular, Kastendieck et al. (2021) deliberately embedded emotional contagion within social contexts to examine the impact of context on emotional contagion and interpersonal distance [27]. In their experiments, participants were exposed to the social contexts of weddings and funerals, and they were then presented with salient facial expressions (sad and happy) (the facial expressions of others in these contexts were obscured). Thus, participants could be exposed to four different experimental conditions, where the expresser either smiled or cried within a wedding or funeral context. Subsequently, participants were asked to report their subjective experiences, their perceptions of the trustworthiness and morality of the expressers, and the psychological distance between the expressers and themselves. Facial electromyography (fEMG) data of the participants were also recorded. The results indicated that emotional expressers who displayed inappropriate emotions (i.e., exhibited pleasant expressions at funerals) elicited more negative responses from the recipients, who viewed them as untrustworthy and immoral. This study provides further evidence that social contexts can influence emotional contagion and reshape it. However, Kastendieck’s work primarily focused on the relationship between social contexts and the facial expressions of the expressers, rather than the impact of social context on emotional contagion itself. Additionally, the social contexts examined in their study (wedding or funeral contexts) have evident emotional attributes, making it challenging to pinpoint whether it is the mere presence of social contexts or the emotional attributes of those contexts that affect emotional contagion.

In summary, although various studies have indirectly or directly suggested the role of social context in emotional contagion, there are still few studies that directly demonstrate how typical social contexts (neutral, everyday life situations) impact emotional contagion. Such studies would provide the foundation for understanding how individuals process and integrate information between social contexts and facial expressions, ultimately shaping their emotional experiences. Therefore, our study aimed to explore the influence of the presence of social context (Experiment 1) and the clarity of social context (Experiment 2) on emotional contagion through two experiments. We adapted and simplified the experimental paradigm used by Kastendieck et al. (2021) [27] for Experiment 1 and Experiment 2, while also recording the subjective experiences of participants [28,29,30]. We hypothesized that emotional contagion occurs regardless of the presence or absence of social context, whether explicit or ambiguous, but the degree of emotional contagion may vary.

In Experiment 2, we also incorporated EEG techniques to investigate the neural mechanisms underlying the influence of social context on the process of emotional contagion. Taking inspiration from the research conducted by Kuang et al. (2021) on the impact of eye orientation on emotional mimicry, we categorized the intensity of emotional mimicry into high and low intensities based on the fEMG amplitude. Subsequently, we examined the differences in EEG activities associated with these two levels of emotional mimicry intensity to reveal the relevant neural mechanisms. The results indicated the involvement of several components of event-related potential (ERP), namely P1, N1, P2, P3, N2, and LPP, in the processing [15]. Hence, we selected these components as references and aimed to investigate their applicability to the process of emotional contagion within a social context.

## 2. Experiment 1

Emotional contagion often occurs within a specific social context shared by emotional expressers and recipients. However, many studies have overlooked or completely disregarded social context, making it challenging for researchers to gain insights into emotional contagion in real-life situations. Consequently, in Experiment 1, we aimed to address the following question: Does the presence of a common social context influence emotional contagion?

Furthermore, within a given social context, emotional information stemming from various factors (such as the social context itself, emotional expressions, and the relationship between them) may impact the emotional experience of a recipient [31]. Therefore, this study also examined whether individual emotions are solely influenced by social contexts or expressions, or both, and explored their interplay.

### 2.1. Method

#### 2.1.1. Participants

To estimate the required sample size of participants, we utilized G*Power 3.1.9.2 and employed repeated ANOVA with a moderate effect size (f = 0.25), significance level (α) of 0.05, and desired power (1−β err prob) of 0.8. Based on these parameters, a minimum of 24 participants was determined to be necessary. Therefore, we recruited 31 undergraduate participants (six males and 25 females) with an average age of 20.87 ± 2.14 years. All participants had normal or corrected-to-normal vision, were right-handed, had no history of brain organic disorders or emotion-related disorders within the 3 months prior to the experiment, and were in a good emotional state one week prior to the experiment. Prior to the commencement of the study, participants were provided with information regarding the requirements and compensation (CNY 100) for their participation and were required to sign an informed consent form. This study received approval from the relevant ethics review committee.

#### 2.1.2. Materials

The social context pictures used in this study were primarily sourced from the internet. These pictures depict common social activities such as cleaning, taking a walk, waiting for a bus or train, shopping, working, studying, and meeting. It was essential for the social context pictures to feature people engaging in these activities. Furthermore, the expressions of all of the characters in the social context pictures were neutral. In cases where the expressions were not neutral, they were intentionally blurred to eliminate the potential impact of other characters’ emotional expressions on the emotional contagion of the target character. We selected a total of 35 social context pictures, all standardized to a size of 640 × 480 dpi. The expression pictures, on the other hand, were obtained from the Tsinghua facial expression database [32]. We selected 105 young Chinese expressions, including 35 actors (17 male actors), who performed positive, negative (sad expressions), and neutral expressions. The sizes of these expression pictures were standardized to 350 × 464 dpi.

Before conducting the experiment, two separate groups of participants were recruited to rate the social context pictures (*n* = 20, Mage = 18.74 ± 1.05 years, males = 11) and the facial expressions (*n* = 15, Mage = 18.67 ± 0.49 years, males = 7). The evaluation criteria included familiarity, emotional valence, and arousal, and a 9-point Likert scale was used. For the selected social context materials, the average emotional valence was 4.97 ± 0.13, arousal was 3.12 ± 0.43, and familiarity was 7.86 ± 0.86. Regarding the selected facial expression materials, the average valence of positive expressions was 6.26 ± 0.45, arousal was 4.38 ± 0.64, and familiarity was 4.6 ± 0.31. The average valence of neutral expressions was 4.71 ± 0.33, arousal was 2.02 ± 0.38, and familiarity was 4.29 ± 0.39. On the other hand, the average valence of negative expressions was 2.41 ± 0.36, arousal was 4.07 ± 0.52, and familiarity was 4.32 ± 0.39.

#### 2.1.3. Procedure

The participants were asked to fill in a positive and negative affect schedule (PANAS) [33], 5 min before Experiment 1 to evaluate their current emotional status and ensure that they were neither extremely negative nor positive. The scale was divided into two PANAS sub-scales, each with ten 5-point items that were adjectives describing an individual’s emotional state (i.e., “interested”).

To eliminate potential interference and allow for a clear distinction between the emotional experiences of participants when exposed to social contexts only, facial expressions only, or expressers’ expressions in social contexts, the experimental procedure was divided into three sessions with a fixed order. The first session (Expression) measured the emotional experiences of individuals when exposed to expressers’ expressions without any social contexts. This session consisted of three random blocks, with each block containing 35 random trials encompassing neutral, positive, and negative expressions, totaling 105 trials. The second session (Context) measured the participants’ experiences when presented with social contexts alone. This session included one block with 35 random trials. In the third session (Contagion), the participants’ emotional responses after observing others’ expressions in a given social context were evaluated. This session comprised three completely random blocks, each containing 35 random trials with positive, negative, and neutral expressions, amounting to 105 trials. Importantly, the same actor and social context did not appear repeatedly within any block.

In the Expression and Context sessions, the experimental procedures were similar except for the material stimuli. First, a 500 ms “+” fixation was presented, following a 2000 ms picture (expression or context), and then the participants were required to evaluate their own subjective experience. In the Contagion session, after a 500 ms “+” fixation, the social context was presented for 1200 ms, and then a facial expression was exhibited for 2000 ms. Finally, the participants were asked to appraise their own emotional experience (from negative to positive, 9 points, 1 = “extremely negative", 5 = “neutral", 9 = “extremely positive”) after witnessing the emotional response of expressers to the given social context. In particular, participants were introduced to imagine themselves being in the given context when the social contexts appeared regardless of the Context and Contagion sessions.

### 2.2. Results

#### 2.2.1. Emotional State before Experiment 1

The results indicated that the positive state of the participants (*M* = 28.355, *SD* = 5.88) was significantly higher than their negative state (*M* = 14.516, *SD* = 4.04, *t*_30_ = 10.4, *p* < 0.001, Cohen’s *d* = 2.65) before Experiment 1.

#### 2.2.2. Whether Emotional Contagion Occurred

As summarized in Table 1, the emotional experiences of the recipients (positive or negative) were affected by the emotional expressers and were consistent with expressers, regardless of the social contexts. This indicated that emotional contagion occurred. However, the neutral expressions may have evoked negative emotional tendencies of the participants when there was no given social context.

#### 2.2.3. Effect of Social Context on Emotional Contagion

As mentioned before, in contrast to general emotion occurrence or arousal, emotional contagion attaches significance to emotional convergence and consistency between emotional expressers and recipients. Therefore, to intuitively show emotional contagion or even anti-emotional contagion, the scores of the subjective experiences of the participants were transformed as follows: the original scores minus “5” were used if the expressers’ expressions were positive or neutral, otherwise “5” minus the original scores was used. Consequently, regardless of the emotional contagion caused by positive or negative expressions, its occurrence could be characterized as a positive value greater than 0, whereas anti-emotional contagion could be characterized as a negative value less than 0. A higher value indicated a higher contagion intensity. After transformation, a repeated-measures analysis of variance (ANOVA) was conducted to discuss the interaction of social context (absent or present) and expression (positive, negative, or neutral) with emotional contagion. If the analysis did not meet Mauchly’s test of sphericity, the results were corrected by Greenhouse–Geisser (the criterion was also applied in all of the analyses).

The results indicated that regardless of positive and negative emotional contagion, contagion intensity was greater in the absence of social context (*MD* _absent−present_ = 0.259, *SE* = 0.051, *p* < 0.001), with a significant main effect of social context, *F* (1, 30) = 25.926, *p* < 0.001, $\eta_{p}^{2}$ = 0.464. In addition, the main effect of expression (*F* (2, 60) = 78.897, *p* < 0.001, $\eta_{p}^{2}$ = 0.714) was found, indicating that regardless of social context, the contagion intensity of positive and negative emotions was similar (*p* = 0.415), both of which were significantly greater than that of neutral expressions (all *p* < 0.001).

**Figure 1:**
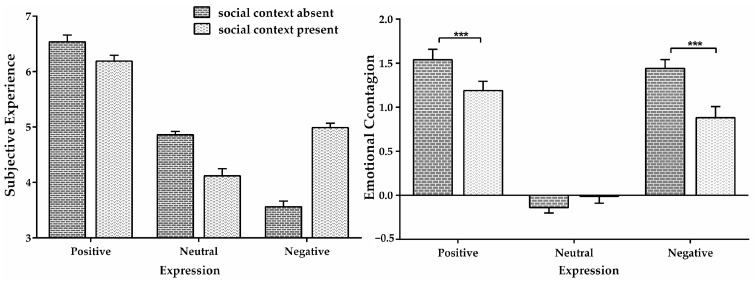
The figure on the left shows the score of emotional experience, and that on the right shows the score of emotional contagion in Experiment 1. The error bar represents the standard error, “***” *p* < 0.001.

Furthermore, the interaction between social context and expression was found (Figure 1), *F* (2, 60) = 12.457, *p* = 0, $\eta_{p}^{2}$ = 0.293; simple effect analysis indicated that for positive and negative expressions, the emotional contagion intensity without social context was significantly greater than that with social context (positive: *MD* _absent−present_ = 0.259, *SE* = 0.088, *p* < 0.001; negative: *MD* _absent−present_ = 0.558, *SE* = 0.124, *p* < 0.001). In contrast, for neutral facial expressions, the presence or absence of social context had little effect on emotional contagion intensity (*MD* _absent−present_ = −1.29, *SE* = 0.067, *p* = 0.065).

### 2.3. Discussion

Although related theories and previous studies have emphasized the role of social context in emotional contagion, there is a lack of research comparing the difference in emotional contagion between the presence and absence of social contexts. Therefore, Experiment 1 aimed to explore this issue. The results of Experiment 1 confirmed the significance of social context in emotional contagion. Regardless of whether positive or negative expressions were observed in a specific social context, the emotional recipients experienced emotions similar to those of the expressers. However, the presence of social contexts had a moderating effect on the contagion, reducing its intensity compared to situations where social contexts were absent. This suggests that in previous studies that neglected social contexts, the intensity of emotional contagion might have been exaggerated. This amplification effect may be more pronounced among individuals or groups who rely heavily on contextual cues to interpret others’ expressions, such as Japanese individuals, who are more influenced by their social background compared to Westerners when judging the emotions of a central character [34].

In particular, contrary to our initial expectations, participants’ subjective experience leaned toward negativity when they were exposed to neutral expressions (control condition) in the absence of social contextual information. However, this pattern did not hold true when social contexts were present. The participants’ responses to neutral expressions in the absence of social context might depend on the availability of contextual information or its ambiguity. Consistent with previous research [35,36,37], our study demonstrated that a neutral facial expression, in the absence of social context, can be perceived as having a negative bias, leading to unpleasant emotional experiences. This perception could be attributed to facial structure, specifically the natural downward orientation of the corners of the mouth, which is often associated with negative emotions [38]. Additionally, it could be influenced by participants’ negative moods [39], cultural context [37], or emotional knowledge [40], causing them to adopt a negative interpretive lens when understanding the world. However, in this study, we excluded the influence of negative mood as participants’ positive emotional states were significantly higher than their negative emotional states before the experiment, as indicated by the PANAS measurements. On the other hand, the presence of social context clarifies the meaning of ambiguous expressions and corrects cognitive biases caused by the aforementioned factors, enabling participants to make more objective judgments. Furthermore, in a safe and neutral context, individuals may not be affected by extremely subtle negative expressions of others, resulting in no significant difference in emotional contagion compared to the absence of a social environment (although this difference is minimal). This could be attributed to the amplification of the lack of effective information. Additionally, differences in attention allocation strategies in the presence or absence of social context may also contribute to changes in emotional contagion. When a social context is present, individuals may divert part of their attention to the surrounding environment to gather additional information to assist with emotional appraisal, potentially weakening the processing of the expresser’s expression. Conversely, in the absence of social context, an individual’s attention is primarily focused on the expresser’s expression, leading to more in-depth processing of emotional expressions [34].

However, emotional contagion in real life is inseparable from social context, as it involves the exchange of emotional information during social interactions within a specific context [9,41]. Emotional contagion that disregards social situational factors represents a simplified and idealized state. However, similar situations can be observed in practical scenarios. For instance, in certain contexts, individuals may lack access to effective sources of information, possess limited knowledge, or have a limited understanding of their social environment, resulting in blocked and restricted transmission of information through the social context to emotional recipients. In such cases, the social context can be ambiguous, similarly to the absence of a social context. In Experiment 1, we adopted a modified paradigm in Session 3 to examine the impact of social context on emotional contagion, which yielded successful results. Therefore, this paradigm was also employed in Experiment 2.

Currently, there is a lack of sufficient research on the influence of the ambiguity of social context on emotional contagion. In Experiment 2, our aim was to address the following questions: Does the outcome align or exhibit similarities to that of Experiment 1 when social contexts are ambiguous? Additionally, we aimed to identify the key neural activities involved in this process.

## 3. Experiment 2

In Experiment 1, we established that ordinary life contexts have an impact on emotional contagion. In reality, emotional contagion cannot be completely separated from social context, and the mode and intensity of contagion vary across different social contexts.

The amount of information obtained from the social context can influence the occurrence of emotional contagion. When the social context is unclear, individuals are more likely to rely on others’ expressions as a source of information and reference. Therefore, in Experiment 2, we aimed to further investigate the effect of the clarity or fuzziness of social context on emotional contagion. We simulated a fuzzy social context to represent limited information and inadequate understanding, while a clear social context represented a situation where individuals could clearly comprehend the emotional background.

Given the high temporal resolution of EEG techniques and the rapid changes in mental processes during emotional contagion, we believe that EEG techniques are suitable for studying the neural mechanisms of emotional contagion in a social context. Furthermore, EEG technology has a solid research foundation in the field of emotional contagion [15], which made it appropriate for exploring the neural mechanisms in Experiment 2.

### 3.1. Method

#### 3.1.1. Participants

Here, 31 students (*M* = 19.87 ± 5.2 years, 15 males) with normal vision or corrected vision, right-handed, no brain organic disorders, no mood disorders within 3 months, and a stable emotional state 1 week before Experiment 2 were recruited. The participants were required to sign an informed consent form before the experiment, and they would receive a CNY 200 reward after the experiment. This study was reviewed and approved by the local ethics review committee.

#### 3.1.2. Materials

The selection criterion for clear social contextual pictures and facial expressions was the same as in Experiment 1. Forty-two clear social pictures were selected; the pictures were blurred via Photoshop to form 42 fuzzy social context pictures. Thus, participants could not effectively recognize the social contextual content depicted in the pictures, and the original color block distribution was retained to balance the interference of visual distribution in EEG acquisition. Finally, 84 context pictures with a unified size of 640 × 480 dpi and 126 facial expressions involving positive, negative, and neutral expressions with a unified size of 350 × 464 dpi were selected.

#### 3.1.3. Procedure

Before the experiment, participants were seated in a separate and quiet laboratory and kept a distance of more than 90 cm from the computer screen. They were then instructed to fill in the PANAS 5 min before the experiment.

**Figure 2:**
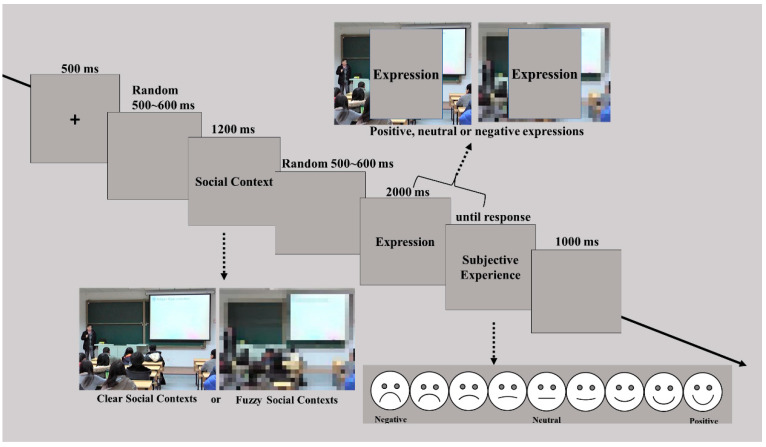
The experimental procedure of Experiment 2.

In the formal experiment (Figure 2), a 500 ms fixation “+” was presented to remind the participants that the target would appear, following a 1200 ms context picture, and then they were simultaneously asked to imagine that they were in such a situation when exposed to the picture. Thereafter, a 2000 ms facial expression was presented on the context picture, and the participants were required to evaluate their own immediate emotional experience after observing the emotional reaction of others to the given social context (9-point rating score, 1 = “extremely unpleasant”, 5 = “neutral”, 9 = “extremely pleasant”). In particular, the participants were instructed that the blurred picture represented a lack of knowledge about environmental information.

There were six random blocks, and each block contained 42 random trials and a 2 min rest interval. The same context was repeated three times, but the subsequent emotional expressions were different; similarly, the same expression was repeated twice, but the previous context was different, and the same context or expression could not appear in the same block.

#### 3.1.4. EEG Data Recording and Analysis

EEG data were collected using a BIOSEMI Active-Two amplifier system [42], and a 64-channel EEG cap was placed on the head according to the International 10–20 system, and EEG data were recorded by BIOSEMI’s data recording software Active 8, with a 1024 Hz online sampling rate, 0.01–417 Hz bandwidth, the Common Modesense (CMS) as the online reference point and the Driven Right Leg (DRL) as grounding (http://www.biosemi/faq/cms&drl.htm, accessed on 18 September 2021). Three external electrodes were attached to record the potential of the left, right mastoid, and right vertical ophthalmic electricity. During the experiment, the voltage at each electrode was maintained below 40 μV.

Preprocessing of the EEG signal was performed using the EEGLAB toolkit under the MATLAB platform [43]. First, by adjusting the off-line sampling rate to 512 Hz, channel location was conducted, and the off-line reference was converted as a bilateral mastoid; thereafter, high-pass and low-pass filterings of 0.1 Hz and 40 Hz, respectively, were implemented. Afterward, the kurtosis method was used to detect the bad electrodes (no more than 4), and the bad electrodes were replaced using an interpolation algorithm. Subsequently, the large artifacts and segments without tasks (such as rest) were manually checked and eliminated, and independent component analysis [43] was performed to reject the ocular artifact components. Thereafter, the EEG data were segmented (−200 ms–1000 ms), the baseline was calibrated (−200 ms–0 ms), and extreme values of more than ±75 μV were eliminated. Finally, EEG data were superimposed and averaged under different experimental conditions. Moreover, if the reserved segments for any condition were less than 30, the corresponding data of the participant were not included in the final analysis. Therefore, four participants were excluded owing to inappropriate data collection, recording, or inadequate retention.

Among the early components of interest, N1, N170, and N2 were mainly considered in this study. For N1, the average amplitude of the prefrontal region was approximately 110 ± 15 ms after stimuli onset was selected, including the FPZ, FP1, FP2, AFZ, AF3, AF4, AF7, and AF8 electrodes. For the N170, the parietal occipital region with an average amplitude of approximately 170 ± 15 ms after stimuli onset was selected, including POZ, PO3, PO4, PO7, PO8, OZ, O1, and O2 electrodes. Similarly to N1, the prefrontal region had an average amplitude of approximately 210 ± 15 ms after stimuli onset, containing the FPZ, FP1, FP2, AFZ, AF3, AF4, AF7, and AF8.

Moreover, for middle and late components of interest, P3 and LPP were mainly considered owing to their close relationship with emotion and higher-order evaluation [44,45]. The parietal, parietal-occipital, and occipital regions were considered for P3, including the PZ, P1, P2, P3, P4, P5, P6, POZ, PO3, PO4, PO7, PO8, OZ, O1, and O2, and their average amplitude of 350 ± 40 ms after stimulus initiation was calculated. In this study, LPP mainly concentrated on the posterior–superior of the brain scalp [46], including the CP1, CP2, CP3, CP4, P1, P2, P3, P4, PO3, and PO4 electrodes. The time window ranged from 400 ms to 1000 ms after the presentation of stimulation, and then it was divided into three sub-time windows of 400–600 ms (early), 600–800 ms (middle), and 800–1000 ms (late); the amplitude was stacked and averaged under the corresponding time window. Finally, behavioral data and EEG data after preprocessing were statistically analyzed using SPSS 22.

### 3.2. Results

#### 3.2.1. Behavior Results

A paired sample *t*-test found that positive experience was significantly higher than negative experience (positive: *M* = 30.58, *SD* = 5.672; negative: *M* = 18.85, *SD* = 6.601), *t* _26_ = 7.079, *p* < 0.001, Cohen’s *d* = 1.89.

As shown in Table 2, consistent with Experiment 1, regardless of social context, both positive and negative expressions would cause participants to experience emotions similar to those of the expressers. Neutral expressions (control condition) did not evoke the recipients’ clear emotional reactions when the social context was clear. In contrast, the participants experienced a slightly negative experience when exposed to a fuzzy social context.

Similarly to Experiment 1, subjective experience scores were converted to a behavioral index of emotional contagion. Thereafter, a 2 × 3 repeated-measures ANOVA was conducted, with social context (fuzzy and clear) and expression (positive, negative, and neutral) as the within-subject independent variables.

A main effect of expression was obtained, *F* (2, 52) = 66.309, *p* < 0.001, $\eta_{p}^{2}$ = 0.718, and post hoc tests indicated that the emotional contagion intensity caused by positive and negative expressions was similar (*p* = 0.768), and both were higher compared to those caused by neutral expressions (all *p* < 0.01). However, no main effect of social context was found, *F* (1, 26) = 0.045, *p* = 0.834, $\eta_{p}^{2}$ = 0.002.

**Figure 3:**
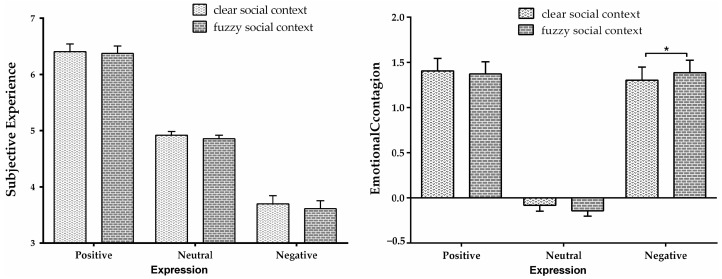
The figure on the left shows the score of emotional experience, and that on the right shows the score of emotional contagion in Experiment 2. The error bar represented the standard error, “*” *p* < 0.05.

Additionally, the interaction between social context and expression (*F* (2, 52) = 3.39, *p* = 0.041, $\eta_{p}^{2}$ = 0.115; Figure 3) indicated that except for the significant difference in emotional contagion of negative expressions for clear and fuzzy contexts (*p* = 0.048), no significant difference between clear and fuzzy social contexts under positive or neutral expressions was discovered (*p* _positive_ = 0.437; *p* _neutral_ = 0.062). Similarly to Experiment 1, Experiment 2 proved that negative emotional contagion was relatively more susceptible to social contexts (than positive emotional contagion).

#### 3.2.2. Results of ERP

**Figure 4:**
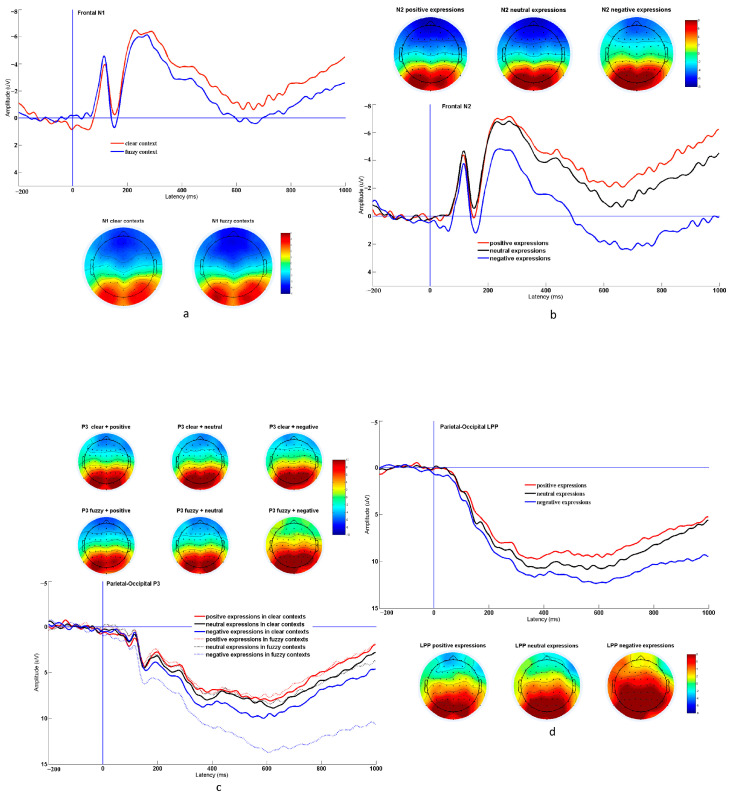
The main results of ERP in Experiment 2. The waveform maps showed the average waves of all electrodes selected for experimental analysis, and the topographic maps showed the activation level of the scalp in the time windows selected for analysis. (**a**) showed the main effect of Context on N1 components; (**b**) showed the main effect of Expression on N2 components, (**c**) showed the interaction between Context and Expression on P3 components, legend “clear” or “fuzzy” referred to clear or fuzzy contexts, respectively, and legend “positive”, “neutral”, or “negative” referred to positive, neutral, or negative expressions of expressers respectively; (**d**) showed the main effect of Expression on LPP components.

The ERP data of the emotional contagion stage were collected as dependent variables, and social context (clear, fuzzy) and expression (positive, neutral, and negative) were taken as independent variables for the repeated-measure ANOVA (Figure 4). Furthermore, the Bonferroni correction was applied when the post hoc test or simple effect test was applied to all ERP data.

For the N1 component, the clear social context was accompanied by a smaller N1 amplitude than the fuzzy social context (*MD* = 0.847, *SE* = 0.310, *p* = 0.011), with a significant main effect of social context, *F* (1, 26) = 7.447, *p* = 0.011, $\eta_{p}^{2}$ = 0.223). Nevertheless, the main effects of expression (*F* (2, 52) = 1.407, *p* = 0.254, $\eta_{p}^{2}$ = 0.051) and its interaction with social context were not significant (*F* (2, 52) = 1.238, *p* = 0.298, $\eta_{p}^{2}$ = 0.045).

For the N170 component, the main effects of social context (*F* (1, 26) = 1.842, *p* = 0.186, $\eta_{p}^{2}$ = 0.066) and expression (*F* (2, 52) = 1.711, *p* = 0.191, $\eta_{p}^{2}$ = 0.062), and their interaction (*F* (2, 52) = 0.767, *p* = 0.47, $\eta_{p}^{2}$ = 0.029) were not identified.

For the N2 component, the main effects of social context (*F* (1, 26) = 0.086, *p* = 0.771, $\eta_{p}^{2}$ = 0.003) and interaction between social context and expression were not found (*F* (2, 52) = 0.174, *p* = 0.84, $\eta_{p}^{2}$ = 0.007). The main effect of expression (*F* (2, 52) = 9.777, *p* < 0.001, $\eta_{p}^{2}$ = 0.273) indicated that the N2 amplitudes induced by neutral and positive expressions were similar (*p* = 0.991), and they were greater than those induced by negative expressions (*p* _positive_ = 0.002, *p* _neutral_ < 0.001).

For the P3 component, there was neither a main effect of social context (*F* (1, 26) = 0.952, *p* = 0.338, $\eta_{p}^{2}$ = 0.035) nor a main effect of expression (*F* (2, 52) = 1.234, *p* = 0.299, $\eta_{p}^{2}$ = 0.045). However, their interaction (*F* (2, 52) = 4.999, *p* = 0.01, $\eta_{p}^{2}$ = 0.161) showed that positive and negative emotional contagion were not affected by the clarity of the social contexts in the P3 component. In contrast, the P3 amplitude brought about by clear social contexts was smaller than that under fuzzy social contexts for neutral expressions.

In addition, for the LPP components, as depicted before, the time window of the LPP was divided and considered as an independent variable to explore its tendency in different time windows. Therefore, the time window (early, middle, and late), social context (fuzzy and clear), and expression (positive, neutral, and negative) were taken as independent variables, and the average amplitude of LPP was taken as the dependent variable in the repeated-measures ANOVA.

The results indicated that as the time window increased, the LPP amplitude decreased gradually (all *p* < 0.01), which was revealed by a significant main effect of time window, *F* (2, 52) = 36.673, *p* < 0.001, $\eta_{p}^{2}$ = 0.585. Moreover, the main effect of expression (*F* (2, 52) = 5.663, *p* = 0.006, $\eta_{p}^{2}$ = 0.179) indicated that the amplitude of LPP under positive expression was lower than that under neutral expression and negative expression (all *p* < 0.05); there was no difference between neutral and negative expressions (*p* = 1). The main effects of the social context (*F* (1, 26) = 1.517, *p* = 0.229, $\eta_{p}^{2}$ = 0.055) and interactions between time window and expression (*F* (4, 104) = 2.014, *p* = 0.098, $\eta_{p}^{2}$ = 0.072), social context and expression (*F* (2, 52) = 0.687, *p* = 0.507, $\eta_{p}^{2}$ = 0.026), the time window and social context (*F* (2, 52) = 1.34, *p* = 0.271, $\eta_{p}^{2}$ = 0.049), and triple interaction (*F* (4, 104) = 0.67, *p* = 0.614, $\eta_{p}^{2}$ = 0.025) were not observed.

### 3.3. Discussion

Consistent with previous expectations, the behavioral outcomes of Experiment 1 were observed in Experiment 2. Regardless of whether the social context was clear or fuzzy, the emotional experience of recipients was affected by the expression of the expresser when sharing social context. However, contagion intensity depends on the characteristics of the social context; when the social context is vague, individuals may be more susceptible to the emotional contagion of others. In particular, the consistency of the results of Experiments 1 and 2 also verified the reliability and stability of the current experimental paradigm.

In contrast to Experiment 2, the associated neural mechanism involving emotional contagion in a specific social context was further discussed, and the results indicated that different ERP components were sensitive to different psychological processes. According to the two-stage model of Schupp et al. [45], emotional stimuli can guide attentional choices in cognitive processing. First, when emotional stimuli appear, a high-capacity perceptual scanning stage is responsible for complete information perception, screening of stimuli, and preliminary classification, which may be reflected in the ERP components, including N1, N170, and N2 in the current experiment. Once an important stimulus is detected, it enters the second stage, in which the cognitive resources are limited and the perception system may summon the cognitive resources required for processing; therefore, the individual can pay attention and consciously recognize the stimulus, which may be reflected in the P3 components. Subsequently, information processing may enter a deep stage of emotional stimuli processing, involving cognitive evaluation, subjective experience, and meaning processing, which may be reflected in LPP.

Specifically, this study suggests that N1 components might be more sensitive to the aftereffects of contextual stimuli, as shown by the main effect of social context. Because contextual stimuli were only slight and marginal information while expression stimuli were located in the visual center and were more salient in the emotional contagion stage, social contextual pictures below emotional expressions were less likely to be captured in the early stage of visual processing. Thus, the difference in the N1 amplitude between different social contexts rather than the expressions could be mainly because of the previous contextual stimuli that caused different psychological expectations of participants toward the upcoming expression stimulus. More specifically, when the participants were exposed to a blurry social context, they may have been more likely to allocate attentional resources to the expression of expressers associated with the environment to attain effective information, promoting adaptive behaviors in the current situation for a better chance of surviving and interpersonal relationships. In contrast, when the participants knew the surrounding environment in advance, they were able to prepare to avoid potential environmental risks, and when they were aware that the situation was relatively safe, social appraisal played a crucial role in promoting interpersonal relationships rather than survival [47,48,49]. Therefore, the motivation to devote attentional resources to the expressions of strangers might weaken and is shown via a relatively weak N1 amplitude. Individuals noticed the faces of expressers and their corresponding expressions. Evidently, the difference in emotional faces did not cause a difference in the N170 components in cognitive processing; however, it was reflected in the subsequent N2 amplitude.

Compared with neutral and positive emotional expressions, individuals who were exposed to negative emotional expressions had a smaller N2 amplitude, specifically in the negative fuzzy contexts. As mentioned previously, prefrontal N2 often appears in stimulus classification tasks [50], and it may reflect the allocation and transformation of attention [51]. Previous studies demonstrated that negative emotional stimuli tend to narrow but concentrate attention and its range to promote the subsequent processing to target faster and more accurately, whereas positive emotions tend to stimulate more attention and broaden attentional range to inspire creativity; however, they increase the processing time [52,53]. In Experiment 2, individuals were required to integrate information originating from the social context and facial expressions. However, when the social contexts were neutral, the advantage of attention attraction of negative expressions led individuals to rarely transfer their attention to the surrounding environmental information. However, for positive and neutral expressions, individuals easily expanded their attention to the surrounding social context and integrated situational information into cognitive processing, thus inducing a larger N2 amplitude.

Many studies stated that P3 is an important stage for information to enter conscious processing [54,55]; its amplitude may be related to the allocation and consumption of psychological resources. In Experiment 2, the amplitude of P3 in the clear social context was significantly smaller than that in the fuzzy social context for neutral expressions, which is consistent with the previous hypothesis. In a clear social context, individuals could obtain more information from the social context in advance, which increases confidence in the sufficient grasp of the context information, and thereby urges the individual to reduce their attention toward obtaining situational information via others’ expressions. However, for positive and negative expressions, as the processing was relatively more complex, a similar effect might be reflected in the continuous LPP of P3b.

With the deepening of conscious processing of emotional stimuli, individuals would integrate the information stemming from facial expressions and social context, balance their relationship, and then form corresponding cognitive appraisal, which plays an important role in predicting individual subsequent subjective experiences and may be reflected by LPP amplitude [44,56]. In this study, the LPP amplitude associated with positive expressions was smaller than that associated with negative and neutral expressions, since pleasant experiences caused by positive expressions may reduce physiological arousal and cognitive involvement related to emotion.

Overall, Experiment 2 discussed the related neural mechanisms of emotional contagion in a given social context and demonstrated that the susceptibility of different ERP components varies with social contexts and emotional expressions. Specifically, in the early stage of emotional contagion, individuals allocate unequal attentional resources to subsequent emotional stimuli according to previous social contexts, and the upcoming stimuli in fuzzy social contexts are allocated more attention. After the extensive scanning stage, unimportant information is filtered, and individuals concentrate on the target (facial expressions) for subsequent detailed processing. At this stage, negative contagion holds certain advantages, as it is easier to attract attention and save cognitive resources. Emotional contagion then proceeds to the stage of conscious processing, and fuzzy social contexts consume more cognitive resources. In further processing related to social evaluation, negative contagion may cause a greater loss of psychological resources. Therefore, it can be inferred that compared with positive contagion, negative contagion more easily attracted attention, whereas it would consume more psychological resources to calm negative emotions in the subsequent in-depth processing.

## 4. General Discussion

Experiments 1 and 2 consistently proved that when emotional expressers and recipients are in an ordinary social context, emotional contagion occurs even if they are strangers. Individuals are highly sensitive to the emotional reactions of others in a social context, as others’ emotional responses are often considered an appropriate or effective action reference for the current context (especially emergencies or fuzzy contexts). Therefore, being consistent with expressers’ emotional reactions is a relatively safe way to deal with the environment, which means social appraisal. As mentioned in the two-way model of emotional contagion [57], social appraisal is one of the two paths that lead to emotional contagion. Social appraisal can also be performed automatically; when individuals perceive themselves in a certain emotional state, knowledge about how people usually react and what impact the emotional response may have on others may be activated and form the basis of social appraisal [58].

Even if emotional contagion could emerge regardless of whether the social context exists, the social context has a special significance in emotional contagion. According to the perspective of emotions as social information theory [48,49], when emotional expressers and recipients are in a common social context, emotion-related information from the social context and others’ expressions is simultaneously processed by emotional recipients. If the shared social context is relatively safe at present and the recipients are well aware of the surrounding environment, when exposed to the expression of the expresser, the emotional convergency may depend on the capability of the recipient for empathy, emotional mimicry, affinity motivation, and social intention. However, when the social context does not exist or is vague, a lack of sufficient understanding of the context information makes emotional recipients lose a sense of control over the surrounding environment, which is unfavorable to individual survival and social communication. Therefore, the appraisal of the environment by others in the environment will become the main source of information for emotional recipients and will become more important. This finding is also supported by social comparison theory [59], which holds that when the context is uncertain, the behavior of others will provide the most reference value.

Although this study proved the role of social context in emotional contagion, some problems still need to be solved. For example, it should be emphasized that the study was mainly aimed at the physical fuzziness of social context rather than psychosocial fuzziness because whether the participants objectively obtain enough information from the environment is not completely equivalent to the fuzziness of the psychosocial environment, but the two enjoy a close relationship. For instance, even if individuals in unfamiliar social or cultural environments obtain enough information from the social context, that may lead to results similar to a lack of physical information owing to the lack of relevant knowledge about what kind of response to the current environment is appropriate. We will further explore this problem in a follow-up study.

Furthermore, the social contexts involved in this study were neutral, and there was no further discussion on the role of emotional attributes (e.g., valence and arousal) of social contexts in emotional contagion. The emotional attributes of the social context and their relationship with emotional expression are also important links for the social context that affect emotional contagion. As mentioned earlier, emotional contagion must occur in a certain social context, and the emotional information received by the emotional receiver has multiple sources, including context, others’ emotional reactions, and multiple relationships involved. For example, Kastendieck et al. (2021) established that when the emotion of social context is mismatched with the expressers’ expression, primitive emotional contagion does not occur, and the evaluation of appropriateness of expressers’ emotional responses to the context mediates the outcomes [27]. However, although this study did not explain the psychological and neural mechanisms behind it further, it suggested that except for the clear and fuzzy attributes of social context, the emotional attributes of social context may have an impact on emotional contagion. Therefore, related problems have been discussed in the relevant research being carried out, and we hope to advance the research further.

## Figures and Tables

**Table 1:** Single sample *t*-test for each condition and value “5” (two-tailed) in experiment 1.

Social Context	Facial Expression	*MD*	*t*	*df*	*p*
Absent	Positive	1.51	14.74	38	0
	Neutral	−0.11	−2.01	38	0.051
	Negative	−1.39	−15.42	38	0
Present	Positive	1.13	11.40	38	0
	Neutral	−0.04	−0.60	38	0.555
	Negative	−0.85	−8.09	38	0

Note: A single sample *t*-test was conducted to examine the difference between the emotional experiences of the participants and a valence score of “5” (representing a completely neutral state). If the difference is significantly greater than 5, it indicates that the emotional state of the participant is positive. Conversely, if the difference is significantly less than 5, it suggests that the participant’s emotional state is negative. If there is no significant difference from 5, it suggests that the participant’s emotional state tends towards neutrality. This analysis provides partial evidence of the occurrence of emotional contagion.

**Table 2:** Single sample *t*-test for each condition and value “5” (two-tailed) in experiment 2.

Social Context	Facial Expression	*MD* (Minus 5)	*t*	*df*	*p*
	Positive	1.373	10.33	26	0
Fuzzy	Neutral	−0.142	−2.31	26	0.029
	Negative	−1.386	−10.07	26	0
	Positive	1.407	10.38	26	0
Clear	Neutral	−0.081	−1.23	26	0.231
	Negative	−1.302	−8.95	26	0

Note: A single sample *t*-test was conducted to examine the difference between the emotional experiences of the participants and a valence score of “5” (representing a completely neutral state). If the difference is significantly greater than 5, it indicates that the emotional state of the participant is positive. Conversely, if the difference is significantly less than 5, it suggests that the participant’s emotional state is negative. If there is no significant difference from 5, it suggests that the participant’s emotional state tends towards neutrality. This analysis provides partial evidence of the occurrence of emotional contagion.

## Data Availability

Study data are available on reasonable request to the corresponding author.
